# Supplementary material for: The genome sequence of the Wainscot Smudge, Ypsolopha scabrella (Linnaeus, 1761)
Source: Wellcome Open Res. 2023 Aug 11;8:341. [Version 1] doi: 10.12688/wellcomeopenres.19837.1 (PMC11249514; doi:10.12688/wellcomeopenres.19837.1)
Supplement: Peer review history

# Reviewer report: Reviewer response for version 1

**DOI:** 10.21956/wellcomeopenres.21969.r86877

**Published:** 2024-07-15

**Authors:** Jerome H L Hui

**Affiliations:** 1 The Chinese University of Hong Kong, Hong Kong, Hong Kong

In this Data Note, Boyes and colleagues sequenced and assembled the genome of moth *Ypsolopha scabrella* (Linnaeus, 1761), commonly known as wainscot hooktip or wainscot smudge. According to the UKmoths and iNaturalist, this species can be found in both England and Wales. Molecular data of this species are scarce prior to this report, and are mainly mitochondrial cytochrome oxidase subunit 1 (COI) gene sequences deposited to the NCBI database. Therefore, this new genome resource will be useful for further studies, ranging from understanding the effect and impact of climate change on them, revealing their population structures, to understanding their evolution with other insects.

 This genome resource is excellent from the summary statistics, with high BUSCO number scores, high sequence continuity, and majority of sequences contained on the 30 pseudochromosomes (plus sex chromosome and mitochondrion). All in all, this is another valuable contribution.

Are sufficient details of methods and materials provided to allow replication by others?

Yes

Is the rationale for creating the dataset(s) clearly described?

Yes

Are the datasets clearly presented in a useable and accessible format?

Yes

Are the protocols appropriate and is the work technically sound?

Yes

Reviewer Expertise:

Genomics, evolution, invertebrates

I confirm that I have read this submission and believe that I have an appropriate level of expertise to confirm that it is of an acceptable scientific standard.

# Reviewer report: Reviewer response for version 1

**DOI:** 10.21956/wellcomeopenres.21969.r74112

**Published:** 2024-07-15

**Authors:** Kai Li

**Affiliations:** 1 Donghua University, Shanghai, China

The genome sequence obtained from *Y. scabrella* will serve as a valuable resource for conducting comparative studies within the Lepidoptera order. The author provides a relatively clear description of sample acquisition, DNA library preparation, high-throughput sequencing, bioinformatics, and other related aspects. Therefore, judging from the key points of the journal review, it is a qualified study, and the relevant sequences are helpful for other researchers.

Are sufficient details of methods and materials provided to allow replication by others?

Yes

Is the rationale for creating the dataset(s) clearly described?

Yes

Are the datasets clearly presented in a useable and accessible format?

Yes

Are the protocols appropriate and is the work technically sound?

Yes

Reviewer Expertise:

Bioinformatics; Insect Molecular Ecology.

I confirm that I have read this submission and believe that I have an appropriate level of expertise to confirm that it is of an acceptable scientific standard.

# Reviewer report: Reviewer response for version 1

**DOI:** 10.21956/wellcomeopenres.21969.r86883

**Published:** 2024-07-15

**Authors:** Feng Zhang

**Affiliations:** 1 Nanjing Agricultural University, Nanjing, China

This manuscript presents a high-quality genome assembly but a low-quality annotation. See detailed comments below:

- “The genome was sequenced from one male *Ypsolopha scabrella* (Figure 1) collected from Wytham Woods, Oxford-shire, UK (51.77, –1.34).”
- Add the ‘E, N’ and measurement unit for the longitude and latitude.
- “The resulting annotation includes 20,761 transcribed mRNAs from 20,594 protein-coding and $NCG non-coding genes.” Explain “$NCG” in detail.
- I don’t think that it is a good idea to annotate the insect genome using BRAKER2, which usually generate more gene models than other pipelines. Those models are often of shorter gene length (mean < 500 aa) and lower BUSCO completeness. In this case, the gene number 20,594 is much higher than those of most lepidopteran published genomes, including world-wide pests. In addition, it doesn't make much sense to annotate the genome while the RNA-seq data is lacking.

Are sufficient details of methods and materials provided to allow replication by others?

Yes

Is the rationale for creating the dataset(s) clearly described?

Yes

Are the datasets clearly presented in a useable and accessible format?

Yes

Are the protocols appropriate and is the work technically sound?

Partly

Reviewer Expertise:

Insect genomics and phylogenomics.

I confirm that I have read this submission and believe that I have an appropriate level of expertise to confirm that it is of an acceptable scientific standard, however I have significant reservations, as outlined above.
